# Arterial Pulse Wave Velocity Signal Reconstruction Using Low Sampling Rates

**DOI:** 10.3390/bios14020092

**Published:** 2024-02-08

**Authors:** Sungcheol Hong, Gerard Coté

**Affiliations:** 1 Department of Biomedical Engineering, Texas A&M University, College Station, TX 77843, USA; 2 Department of Electrical Engineering, Texas A&M University, College Station, TX 77843, USA; 3 Center for Remote Health Technologies and Systems, Texas A&M Engineering Experiment Station, Texas A&M University, College Station, TX 77843, USA

**Keywords:** Nyquist–Shannon sampling, signal reconstruction, biosignal processing, Pulse Wave Velocity, bioimpedance, cardiovascular health

## Abstract

Pulse Wave Velocity (PWV) analysis is valuable for assessing arterial stiffness and cardiovascular health and potentially for estimating blood pressure cufflessly. However, conventional PWV analysis from two transducers spaced closely poses challenges in data management, battery life, and developing the device for continuous real-time applications together along an artery, which typically need data to be recorded at high sampling rates. Specifically, although a pulse signal consists of low-frequency components when used for applications such as determining heart rate, the pulse transit time for transducers near each other along an artery takes place in the millisecond range, typically needing a high sampling rate. To overcome this issue, in this study, we present a novel approach that leverages the Nyquist–Shannon sampling theorem and reconstruction techniques for signals produced by bioimpedance transducers closely spaced along a radial artery. Specifically, we recorded bioimpedance artery pulse signals at a low sampling rate, reducing the data size and subsequently algorithmically reconstructing these signals at a higher sampling rate. We were able to retain vital transit time information and achieved enhanced precision that is comparable to the traditional high-rate sampling method. Our research demonstrates the viability of the algorithmic method for enabling PWV analysis from low-sampling-rate data, overcoming the constraints of conventional approaches. This technique has the potential to contribute to the development of cardiovascular health monitoring and diagnosis using closely spaced wearable devices for real-time and low-resource PWV assessment, enhancing patient care and cardiovascular disease management.

## 1. Introduction

Pulse Wave Velocity (PWV) analysis is a critical and non-invasive technique used in clinical practice and research to assess arterial stiffness [1,2,3] and cardiovascular health [4,5,6]. PWV provides essential insights into the functioning of the arterial system, offering valuable information about arterial compliance, vascular tone, and cardiovascular risk [7,8]. It is a strong predictor of cardiovascular events and overall mortality, making it an indispensable tool for the early detection and management of cardiovascular diseases [9]. However, PWV analysis for closely spaced transducers typically requires high sampling rates, more than 10 kHz, to accurately capture the fast-changing dynamics of arterial pulse wave pairs [10]. Additionally, such high sampling rates come with several challenges, including data management complexities, increased memory storage requirements, limited real-time applications [11,12], and affected power consumption [13,14]. Effectively, the use of high sampling rates can be impractical for wearable devices, which are becoming increasingly popular for continuous health monitoring [15,16].

To address the challenges associated with high-frequency sampling in a wearable device, we propose a novel approach that enables PWV analysis from low-sampling-rate data while maintaining accuracy and precision. Our method leverages the Nyquist–Shannon sampling theorem, a fundamental principle in signal processing, to reconstruct pulse signals at a higher sampling rate [17,18].

## 2. Materials and Methods

In this study, we employed a BIOPAC NICO-100C system to record bioimpedance artery pulse signals along the radial artery of the wrist at reduced sampling rates of 250 Hz, 500 Hz, and 1 kHz. We then reconstructed the pulse signals at a higher sampling rate of 10 kHz using MATLAB. The reference bioimpedance signals were directly recorded at a high sampling rate of 10 kHz using the BIOPAC NICO-100C system and used for comparison to the existing PWV measurement method [19].

### 2.1. Measurement Setup for Radial Artery Pulse Using Bioimpedance

**Figure 1:**
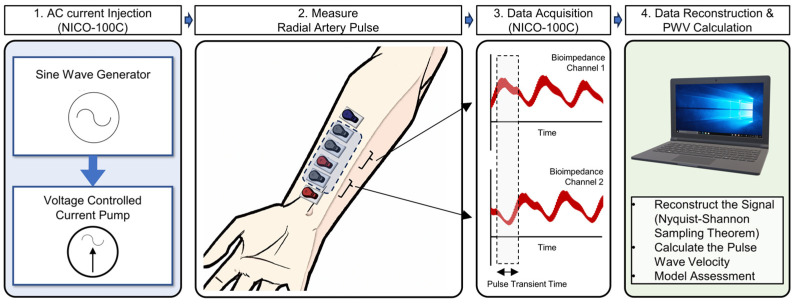
Schematic for measuring radial artery pulse using bioimpedance and the PWV measurement process.

The measurement of the radial artery pulse using bioimpedance and the process of PWV assessment are depicted in Figure 1. To begin, a current pump is utilized to inject a constant sinusoidal current into the wrist, independent of the resistance value of the targeted area [20,21]. This current injection is performed through two electrodes located on the distal and proximal ends, which serve as the injection points for the differential signal. The remaining four electrodes are positioned in between these outer electrodes, two closer to the proximal injection electrode (Channel 1) and two closer to the distal injection electrode (Channel 2), and are used to measure the impedance returning from the tissue and radial artery.

To record the data and carry out this analysis, we employed the BIOPAC NICO-100C system. The acquired data were processed using a custom MATLAB code specifically designed for signal resampling. By reconstructing the pulse signals at a higher sampling rate through resampling techniques, we ensured that vital information was retained and the precision of the PWV measurements was enhanced.

The key step in the PWV analysis was the calculation of pulse transit time (PTT). For this paper, we define PTT as the time taken for the arterial pulse wave to travel between Channel 1 and Channel 2. Once PTT is determined, it is divided by the distance between the channels to extract the PWV [22,23].

#### 2.1.1. Hardware Setup Configuration

**Figure 2:**
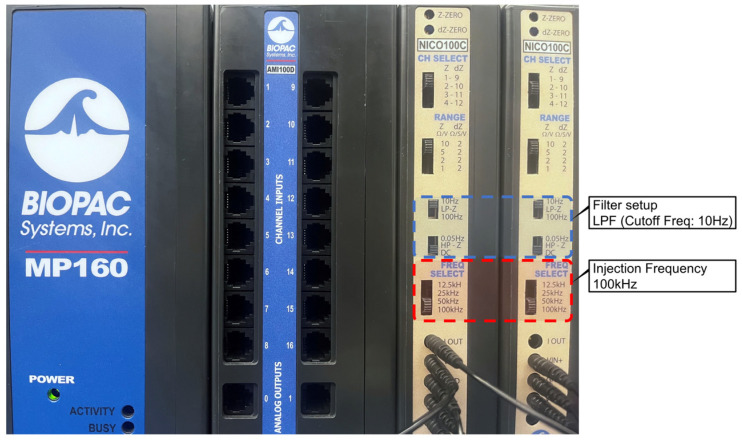
Hardware setup configuration for PWV measurement (injection current frequency of 100 KHz; filter settings included a 10 Hz low-pass filter (LPF)).

For the hardware setup, we utilized the BIOPAC MP160 Data Acquisition and Analysis system, along with two NICO-100C devices [24]. The MP160 was employed for data acquisition and analysis, while the two NICO-100C devices were used for current injection and pulse measurement. Only one of the NICO-100C devices was responsible for current injection, and the second one was connected solely to the voltage measurement terminal [19]. In general, higher frequencies can result in better current conduction through the body’s tissues. Taking this into consideration, we opted to use the highest injection frequency available, which is 100 kHz [24,25,26]. A low-pass filter (LPF) with a cutoff frequency of 10 Hz was also utilized for filtering to eliminate high-frequency noise from the recorded signals. The specific illustration of the hardware configuration is illustrated in Figure 2.

#### 2.1.2. Software Setup Configuration

In the software setup, we used a total of six channels for signal processing. The first and second channels consisted of the raw data recorded at a low sampling rate. To make it easier to visualize the pulse peaks, we created the third and fourth channels to represent the derivative of the raw data. Additionally, to remove the noise and artifacts present in the signals, we applied windows and digital filters to the fifth and sixth channels. For windowing, we opted for the Blackman window, which exhibits reduced sidelobes and higher-frequency resolution compared with classical windows. By utilizing the Blackman window, we effectively minimized spectral leakage [27,28,29], which is vital for obtaining precise pulse wave information. Furthermore, we incorporated a digital low-pass filter with a cutoff frequency of 10 Hz. This filter was applied to each signal to eliminate high-frequency noise. All the sampling rates for each channel were set to the same respective values and were higher than 125 Hz to avoid aliasing in the reconstructed signal [30,31]. Thus, when using the proposed method, it is crucial to identify noise sources in the signal and set the sampling rate to at least higher than the Nyquist frequency of that signal. The software configurations for this signal adjustment can be found in Supplementary Figure S1.

#### 2.1.3. Data Acquisition

For data acquisition, a total of 6 repositionable electrodes were utilized. Two outer electrodes delivered a sinusoidal current of 400 μA with a frequency of 100 kHz [24], while the inner electrodes recorded the voltage variation over a specific time frame and were separated by 3.2 cm. The data were collected for roughly 19 s. The collected data were extracted in the Excel file format to facilitate signal processing.

### 2.2. Biosignal Reconstruction and Processing

**Figure 3:**
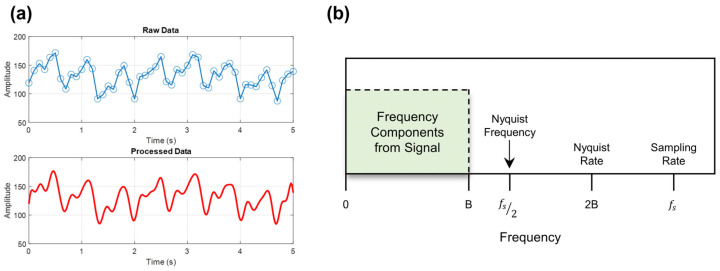
(**a**) Example waveforms of the digitally sampled signal and its reconstructed signal. (**b**) The relationship between Nyquist frequency, Nyquist rate, and the determination of signal frequency and sampling rate.

The signal reconstruction process was carried out using MATLAB. By leveraging the Nyquist–Shannon sampling theorem, we resampled the raw low-sampling-rate data at a higher sampling rate while preserving critical information [31,32]. Visual explanations of these concepts are discussed in Figure 3. The resampling technique allowed us to enhance the precision of the PWV measurements and achieve comparable results with those obtained with the conventional high-sampling-rate method (Figure 3a). The relationship between the sampling frequency threshold and the Nyquist rate is shown in Figure 3b. Where B is the highest frequency component, T = 1fs and $f_{s}$ is the sampling frequency. The sampled signal, $x_{raw}$, can be completely identified from its coordinates at a series of locations separated by fewer than 1/(2B) seconds if it does not contain any frequencies over B Hz. Therefore, anything greater than 2B Hz samples per second is a theoretically sufficient sampling rate. Thus, flawless reconstruction is attainable for a band limit, B<fs2, for a given sample rate, $f_{s}$ [18,31]. In the experimental code used, the raw data’s sampling rate w automatically extracted when the data are inputted. The user then determines the desired sampling rate for signal reconstruction. Subsequently, frequency domain analysis is conducted to verify whether the signal has been correctly interpreted [33,34,35,36].

### 2.3. Data Validation

The data validation process employed in this study followed the established procedures commonly used for PWV analysis [19]. To ensure the reliability and accuracy of our results, we carefully examined the collected data. First, any abnormal data points that deviated significantly from expected physiological ranges were excluded from further analysis. Specifically, we defined significant abnormal deviation in this experiment as data points with PWV values exceeding 20 m/s or falling below 0 m/s, and these were removed from the dataset [23]. Next, we calculated the average PWV value from the remaining valid data points.

## 3. Results

### 3.1. Signal Reconstruction Results

**Figure 4:**
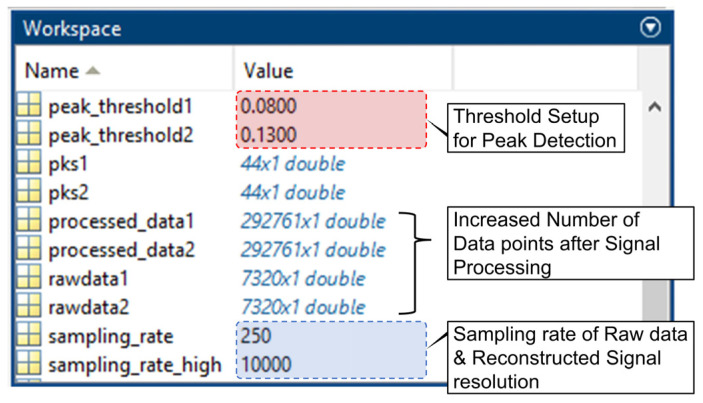
MATLAB Workspace window for verifying signal replication.

The results of the signal reconstruction in the MATLAB Workspace showed a change in frequency and an increased number of samples (Figure 4).

**Figure 5:**
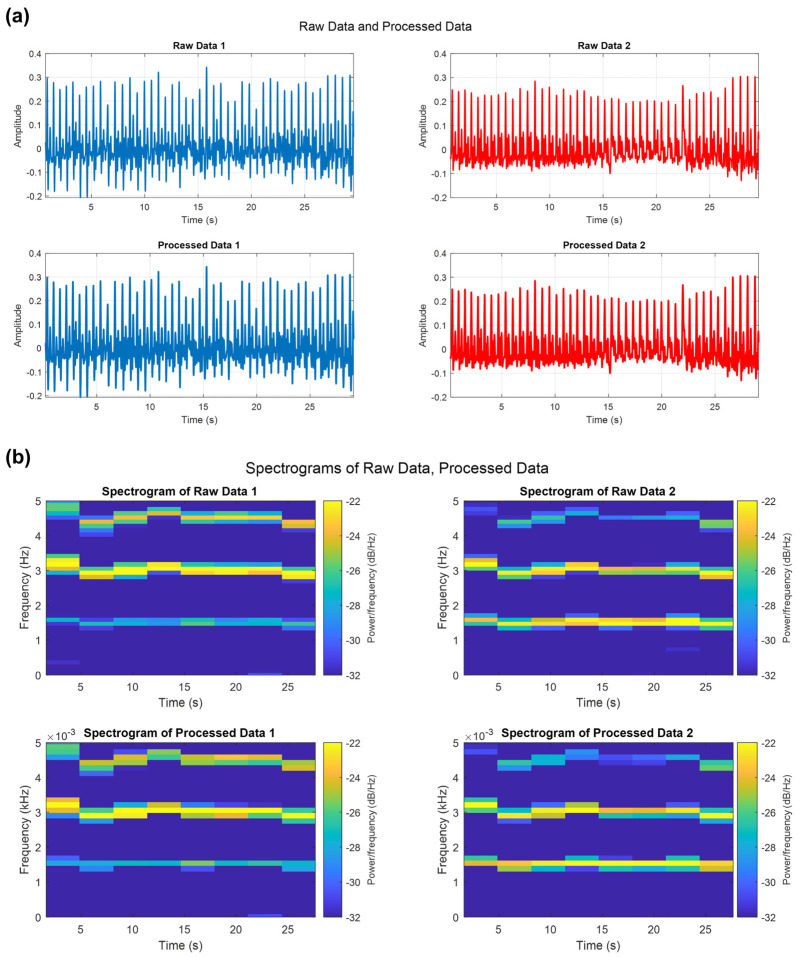
(**a**) Image of representative amplitude data as a function of time for the raw data sampled at 10 KHz; data sampled at 250 Hz; and post-processed, up-sampled 250 Hz data. (**b**) Raw and post-processed data were analyzed in the frequency domain with 5 s divisions. This analysis confirmed that both signals were distributed in the same frequency band throughout the entire time without aliasing.

In addition to utilizing the MATLAB Workspace, the confirmation of signal reconstruction can also be achieved through signal morphology and frequency analysis (Figure 5a,b). In Figure 5a, for this experiment, time is represented on the X-axis, while the Y-axis displays the amplitude of the signal, enabling a direct comparison of the raw and processed signal waveforms. By visually examining the similarity of the original signal and the processed data on the time axis, we could see qualitatively that the signals were similar after the signal reconstruction process. Furthermore, the frequency domain analysis could be used to assess the quality of the signal reconstruction. By comparing the frequency components at the same time intervals, we could further validate the accuracy of the reconstructed signal. Figure 5b qualitatively illustrates the similarity between the raw data and the post-processed data in the frequency domain. Both signals exhibited similar frequency components within the typical heart rate range of 1 to 5 Hz, along with their respective second and third harmonic components [37,38,39]. The consistency of these frequency components confirms the successful reconstruction of the signal, adding further confidence to the reliability of the results. Restored images for other frequencies can be found in Supplementary Figures S2–S4.

**Figure 6:**
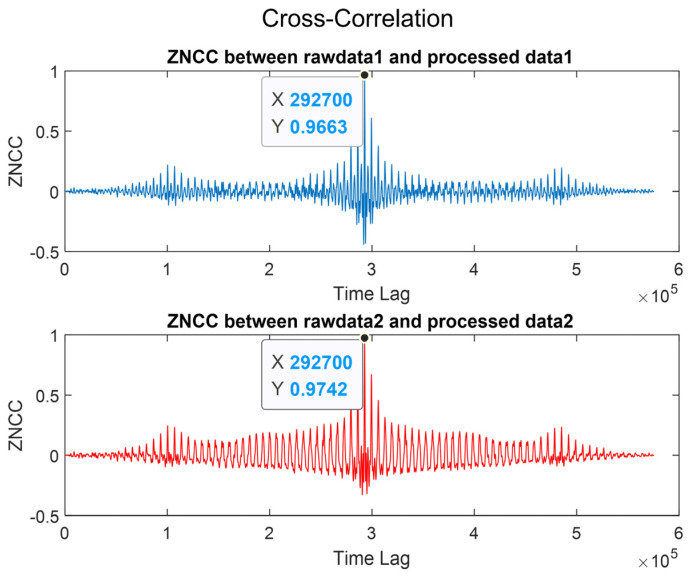
Comparison of signal reconstruction validity using Zero-Normalized Cross-Correlation (ZNCC) between raw data and processed data.

The resemblance of the waveforms served as a robust indicator of the accuracy and efficacy of the reconstruction method employed. As one can see from Figure 6, we investigated the degree of matching using Zero-Normalized Cross-Correlation (ZNCC) [40,41]. All signals exhibited a correlation of 95% or higher, confirming the quantitative similarity between the originals and the signal reconstructions in this experiment.

The use of both time-domain and frequency-domain analyses offered a comprehensive and thorough evaluation of the signal reconstruction process. This dual approach allowed us to confirm the accurate restoration of the original signal, providing insights into the fidelity of the reconstruction method employed. By qualitatively and quantitatively assessing the results from both analyses, we could ascertain the robustness of the signal reconstruction process and the reliability of the data obtained.

**Figure 7:**
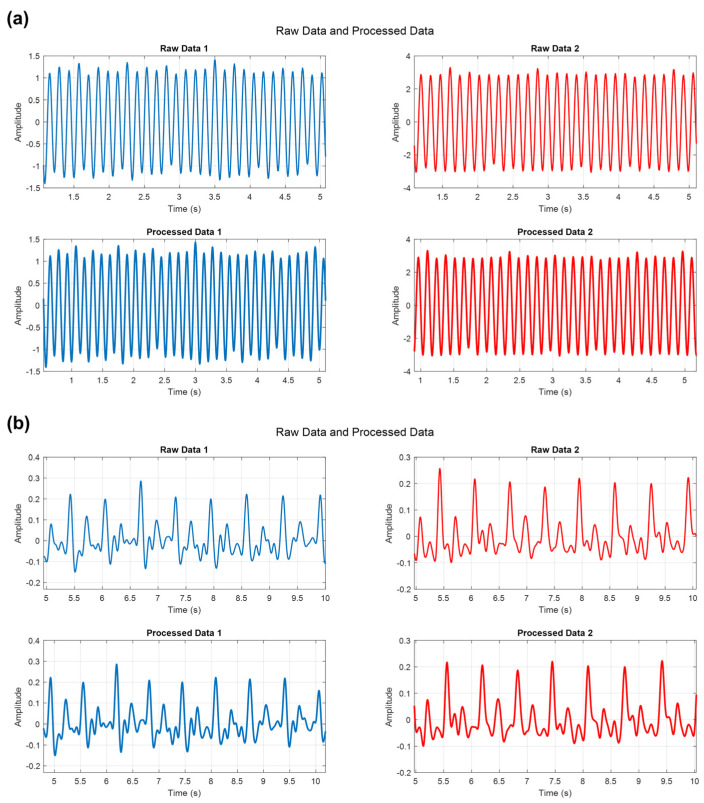
(**a**) The result of signal reconstruction with frequencies sampled below the Nyquist frequency at 125 Hz, showing aliasing from 60 Hz noise. (**b**) Result of a reconstructed waveform above the Nyquist rate at 250 Hz.

During signal reconstruction, there can be numerous conducted or radiated noise sources that may affect the signal [42]. These noise sources can affect the signal frequency component and impact the Nyquist frequency [43,44]. Specifically, in the context of this experiment, when a wall-mounted power source at 60 Hz was used as a noise source and the sampling rate was set to 125 Hz, it resulted in strong aliasing when reconstructing the signal was attempted (Figure 7a). On the other hand, when the signal was properly reconstructed at the Nyquist frequency (250 Hz), a clear pulse was observed (Figure 7b).

### 3.2. Resolution Comparison with the Traditional High-Frequency Sampling Method

To compare the resolution and precision of the proposed method, a pair of bioimpedance signals was recorded at a 10 kHz sampling rate. These signals were then down-sampled to 250 Hz and subsequently reconstructed to 10 kHz using the proposed method. A comparison was performed of these three signals: the down-sampled signal at 250 Hz, the signal reconstructed using the proposed method, and the original 10 kHz signal.

**Figure 8:**
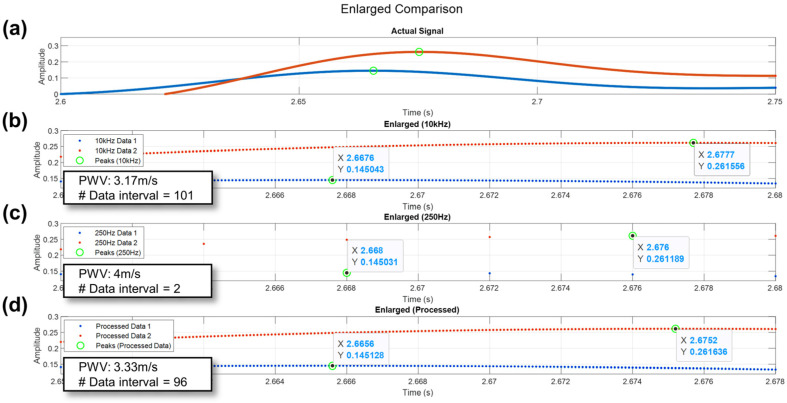
Comparison of resolution for each method based on a pair of pulses. (**a**) Actual signal pair. (**b**) Enlarged view of the data points near the peak for the actual signal (10 kHz). (**c**) Enlarged view of the data points near the peak without processing (250 Hz). (**d**) Enlarged view of the data points near the peak (processed as 10 kHz).

Figure 8a provides an example of the selected signal pairs sampled at 10 kHz, and Figure 8b shows an enlarged version of Figure 8a. In Figure 8c, the enlarged, down-selected 250 Hz signal is depicted, and it can be observed that while the peak points were accurately detected, the data points between the two peak points are limited to only one, indicating a low resolution. However, in Figure 8d, not only have both peak points been successfully identified but also, there are 96 data points between the peaks, which is comparable to the existing method shown in Figure 8b. This demonstrates that the proposed method achieves nearly identical resolution compared to the conventional method. Furthermore, considering that the number of data points between intervals did not simply increase by a factor of 40 (10 kHz divided by 250 Hz), which would have been the case if it were a simple interpolation, but rather by a more complex ratio, it became evident that this was not a result of simple interpolation but rather of the signal having been resampled and reconstructed. This is because the digitized signal and the restored signal had different peak values at different time instances. In fact, when looking at the PTT intervals, it can be seen that the restored signal is more similar to the PTT of the originally recorded 10 kHz signal. The method of peak detection in the restored signal is detailed in Supplementary Figure S5.

The reason for the digitized signal and the restored signal having different peak values can be more easily understood by examining the signal reconstruction method. The reconstruction of a signal is crucial in determining how the signal behaves between discontinuous points. To achieve this, a smoothing factor can be applied. However, this will affect the signal in the time domain, directly impacting the maximum frequency. Hence, applying a smoothing factor in the time domain can result in limiting the signal’s bandwidth to unexpected intervals. The reason for this is that there is a one-to-one correspondence between the time domain and the frequency domain. In this situation, the smoothing factor will result in the neglect of considerations in the frequency domain [18]. This will force a bandwidth restriction, and therefore, we need a method of reconstructing the signal while considering the signal from a frequency-domain perspective. To achieve continuous signal reconstruction from digitized samples, there must be a unique match between the appearances of the sampled signal in its continuous time domain and its frequency domain. The detailed theoretical justification of why the digitized signal and restored signal will have different peaks is provided in the supplemental information, including Supplementary Figure S6 and Supplementary Information. In summary, it is a result of how functions interacting in the convolution process causes peak points to differ between digitized and reconstructed signals. Overall, the result was that this process increased the accuracy in measuring the peak points of pulses, allowing for a more precise measurement of PWV.

### 3.3. PWV Measurement Results

The results of PWV measurement were obtained using the proposed method and compared to the conventional approach across eight trials [19]. The PWV values derived from the proposed method were evaluated to determine if they fell within the normal range [23]. Deviation analysis was also conducted to assess the data dispersion between the two methods. Furthermore, the total number of pulse pairs was examined to identify any abnormal data that could potentially lead to inaccurate results. Cases where data were deemed abnormal (e.g., defined above as data points with PWV values exceeding 20 m/s or falling below 0 m/s) were excluded from this analysis, and the proportion of omitted data was calculated to assess the data fidelity.

**Figure 9:**
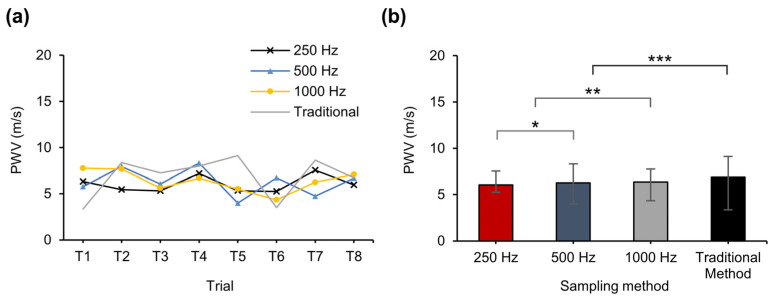
(**a**) PWV values obtained from eight trials using the proposed method and the conventional method. (**b**) The average PWV value for each method. A two-sided unpaired *t*-test was used for statistical data analysis; * *p* = 0.71; ** *p* = 0.70; *** *p* = 0.46.

Each method was tested with eight trials across three sampling frequencies and the traditional 10 KHz sampling, and the results are presented in Figure 9a. According to a published paper that conducted a comprehensive study of 780 healthy individuals, focusing on PWV, it has been revealed that PWV values exhibit a substantial association with age. In the current study context, the anticipated PWV range for ages 30–39 was within the 4.08 m/s to 8.26 m/s bracket, while the total range from the whole participation was 3.12 m/s to 13.4 m/s [45]. Therefore, in this study, we evaluated the effectiveness of our approach based on whether it fell within the PWV range mentioned above. As shown in Figure 9b, the proposed method yielded average PWV values of 6.04 m/s at 250 Hz, 6.27 m/s at 500 Hz, and 6.35 m/s at 1000 Hz, and the conventional method at the 10 kHz sampling rate resulted in 6.87 m/s. These values suggest that the proposed method aligns with the standards of healthy men in their 30s from previous studies. The average PWV for each method showed a comparable accuracy, and the maximum deviation in the average PWV over the eight trials for each method demonstrated comparable accuracy to the conventional method.

**Figure 10:**
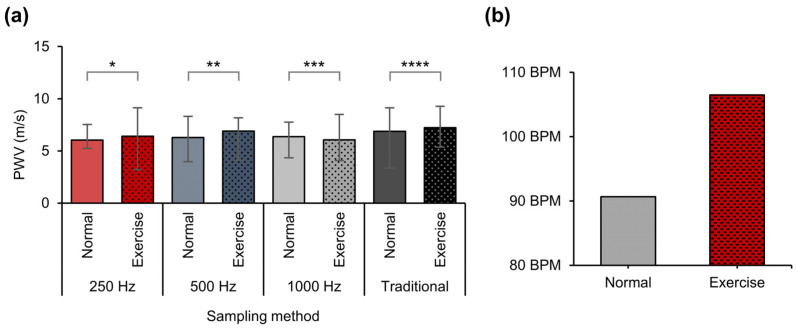
(**a**) Average PWV values before and after exercise from eight trials using the proposed method and the conventional method. A two-sided unpaired *t*-test was used for statistical data analysis; * *p* = 0.65; ** *p* = 0.39; *** *p* = 0.68; **** *p* = 0.70. (**b**) Comparison of heart rates before and after exercise. BPM before exercise = 90.68; after exercise, BPM = 106.5.

In Table 1 and Figure 10a, we have compared the pre- and post-exercise PWV measurements as a function of sampling frequency using the same method to observe what happened to the PWV values with exercise. Considering the fact that PWV is a variable associated with arterial stiffness and that the pressure did not change, it is not surprising that there were no significant changes in the PWV immediately after biking [46]. The participants rode a bicycle at maximum speed for about 10 s. The measurements after exercise were also repeated eight times for each method. As noted, the average PWV value after exercise for the 250 Hz case was 6.41 m/s; for 500 Hz, it was 6.89 m/s; for 1000 Hz, it was 6.06 m/s; and finally, for the conventional 10 kHz, it appeared as 7.23 m/s, showing that there were no significant differences between these and the PWV values before exercise. Additionally, a comparison of heart rates before and after exercise was conducted to verify whether artery pulse signal measurements were performed correctly. Before exercise, the average rate was 91.3 BPM (Beats Per Minute), and after exercise, it was 106.5 BPM. This comparison indicates that, although the PWV did not change significantly, the exercise heart rates did increase and were within the normal range for light exercise, as anticipated [46]. This also aligns with previous research that has suggested that there is no significant relationship between heart rate and PWV [47] and that the proposed method can be applied reliably with variations in heart rate. To cross-verify that the experimental method did not affect blood pressure, blood pressure and heart rate were measured using Finapres^®^ NOVA before and after the bicycle was ridden (Supplementary Figure S7).

Comparisons can also be made from the perspective of data size reductions. All data comparisons were based on roughly 19 s of recorded data. The size of these data at a sampling rate of 250 Hz was 422 KB; at 500 Hz, it was 845 KB; and at 1000 Hz, it was 1.6 MB. The conventional method of PWV measurement at 10 KHz exceeded 16.6 MB of data. This shows why the conventional method poses challenges for real-time wearable device development. Not only does such a large data size impose limitations on hardware for prolonged recording but it also becomes impractical to store. Of course, depending on the added channels and precision ratio, the data size can vary. However, the fact remains that higher sampling rates have the most significant impact on file size. This presents a major obstacle for real-time data transmission. In such situations, high-capacity real-time data processing can potentially lead to data loss. Therefore, data recovery at low sampling rates can have an advantage in terms of hardware and mitigating data loss [48,49,50].

## 4. Discussion

The successful application of the Nyquist–Shannon sampling theorem and signal reconstruction techniques in enabling PWV analysis from low-sampling-rate data has significant implications for the field of cardiovascular health monitoring with wearable devices. In this paper, a performance evaluation was conducted at a sampling rate of 10 kHz in order to conduct comparison using BIOPAC. However, beyond the comparison via BIOPAC, further research is needed on a wider range of PWVs across varying aged subjects to verify the optimal reconstruction sampling frequency for PWV values and show clinical feasibility. By overcoming the constraints of conventional high-sampling-rate approaches, our method opens up new possibilities for PWV collection and analysis using wearable devices. This could lead to more widespread adoption of PWV assessment, enabling the early detection and management of cardiovascular diseases and providing timely and valuable cardiovascular health information to individuals and healthcare professionals. Low-data-management complexities and reduced processing requirements also make our approach well-suited for resource-constrained environments, improving healthcare access in underserved areas.

## Figures and Tables

**Table 1:** PWV values compared before and after exercise.

Sampling Rate	250 Hz		500 Hz		1000 Hz		Traditional	
Conditions	Normal	Exercise	Normal	Exercise	Normal	Exercise	Normal	Exercise
PWV [m/s]	6.04	6.41	6.27	6.89	6.35	6.06	6.87	7.23

## Data Availability

The data presented in this study are available on request from the corresponding authors.

## Supplementary Materials

The following supporting information can be downloaded at: https://www.mdpi.com/article/10.3390/bios14020092/s1.
