# Supplementary material for: Arterial Pulse Wave Velocity Signal Reconstruction Using Low Sampling Rates
Source: Biosensors (Basel). 2024 Feb 8;14(2):92. doi: 10.3390/bios14020092 (PMC10887207; doi:10.3390/bios14020092)
Supplement: Supplementary file 1 [file biosensors-14-00092-s001.zip › biosensors-2776875-supplementary.pdf]

Supplementary Figure 1.

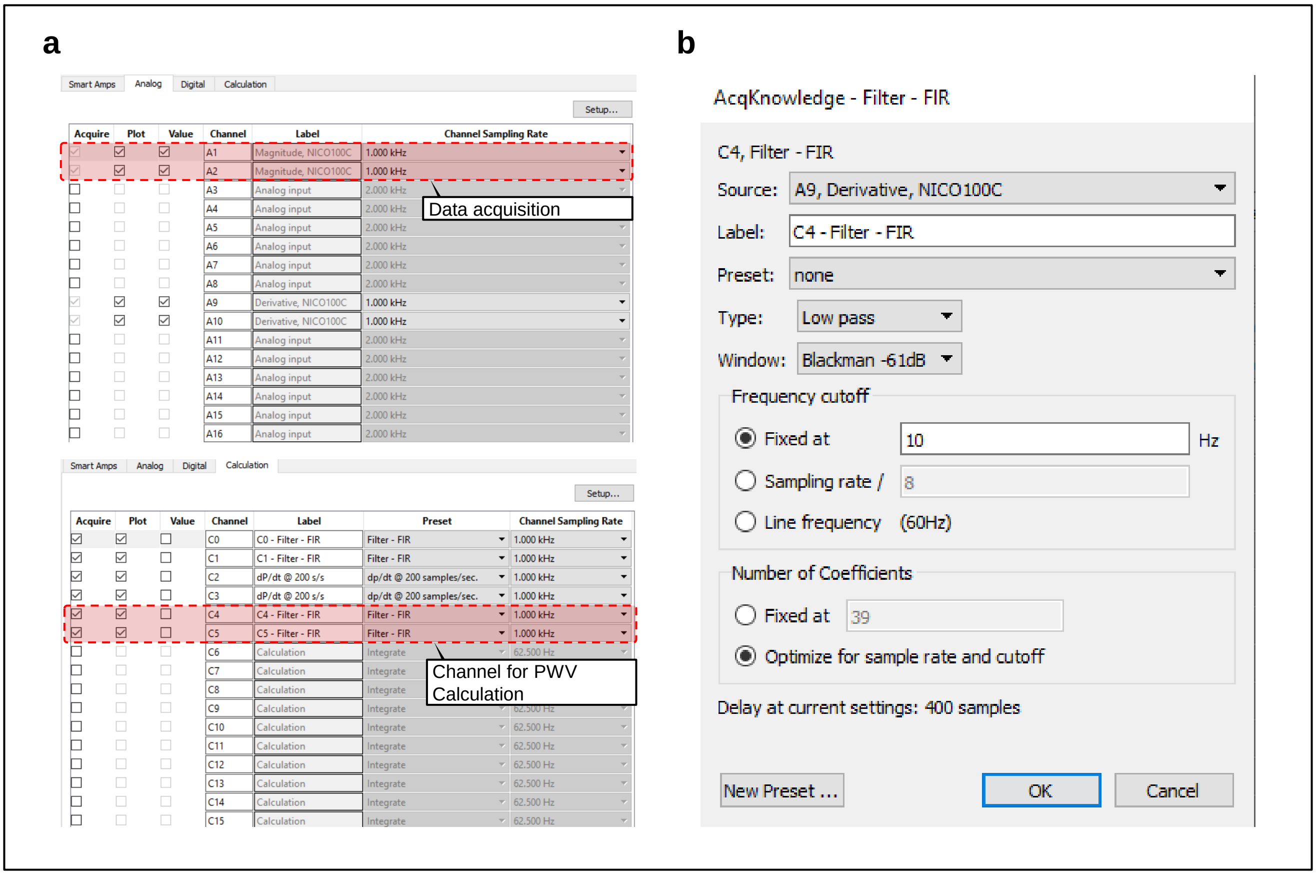

Supplementary Fig.1. The BIOPAC setup for this study. **a.** The sampling rate for the original signal and the differentiated signal for PWV calculation. **b.** Window function and digital filter settings for signal processing.

Supplementary Figure 2.

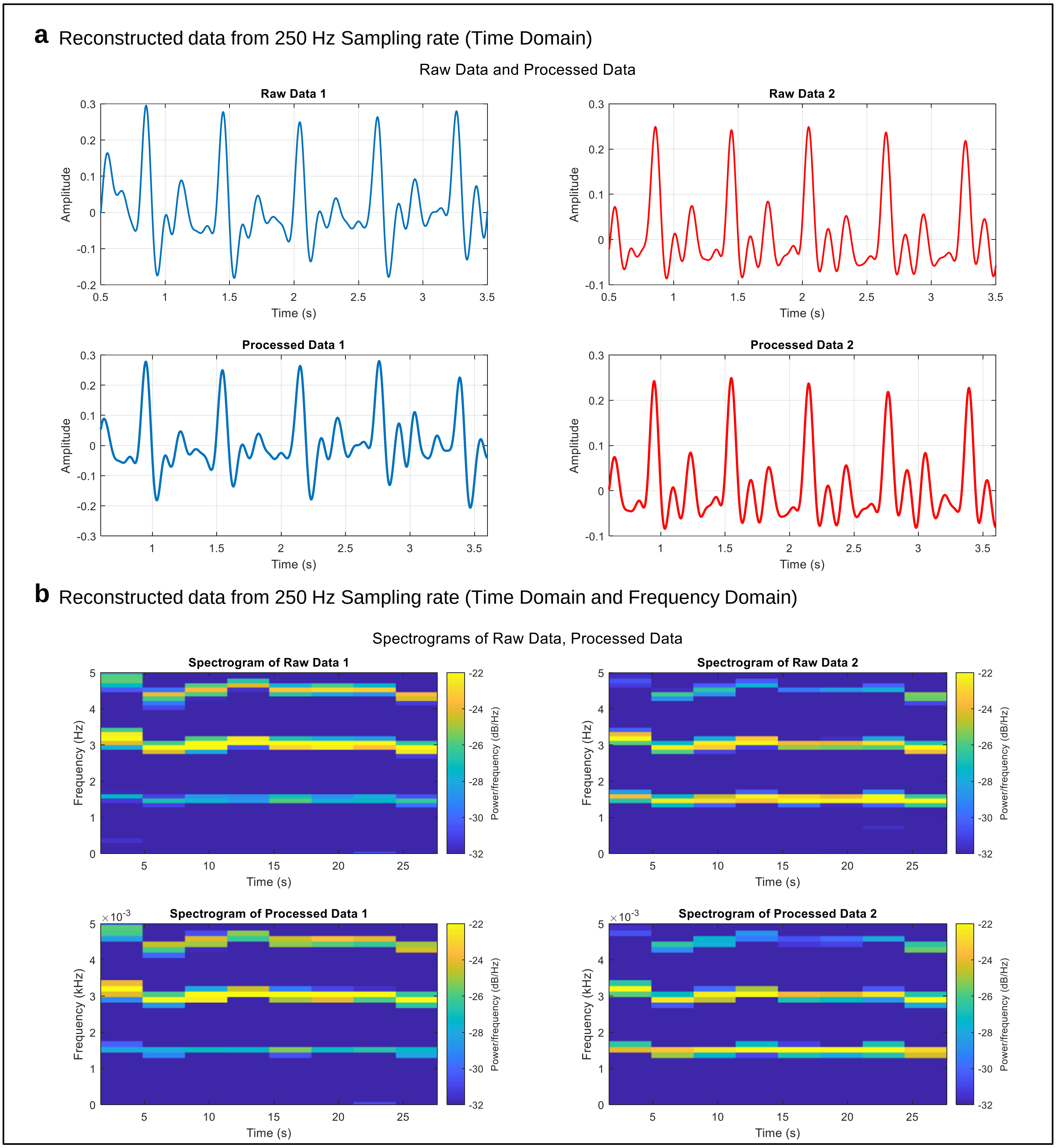

Supplementary Fig.2. Raw signal and reconstructed signal comparison (250 Hz). **a.** Reconstructed data from 250 Hz Sampling rate (Time Domain). **b.** Reconstructed data from 250 Hz Sampling rate (Time Domain and Frequency Domain)

Supplementary Figure 3.

a Reconstructed data from 500 Hz Sampling rate (Time Domain)

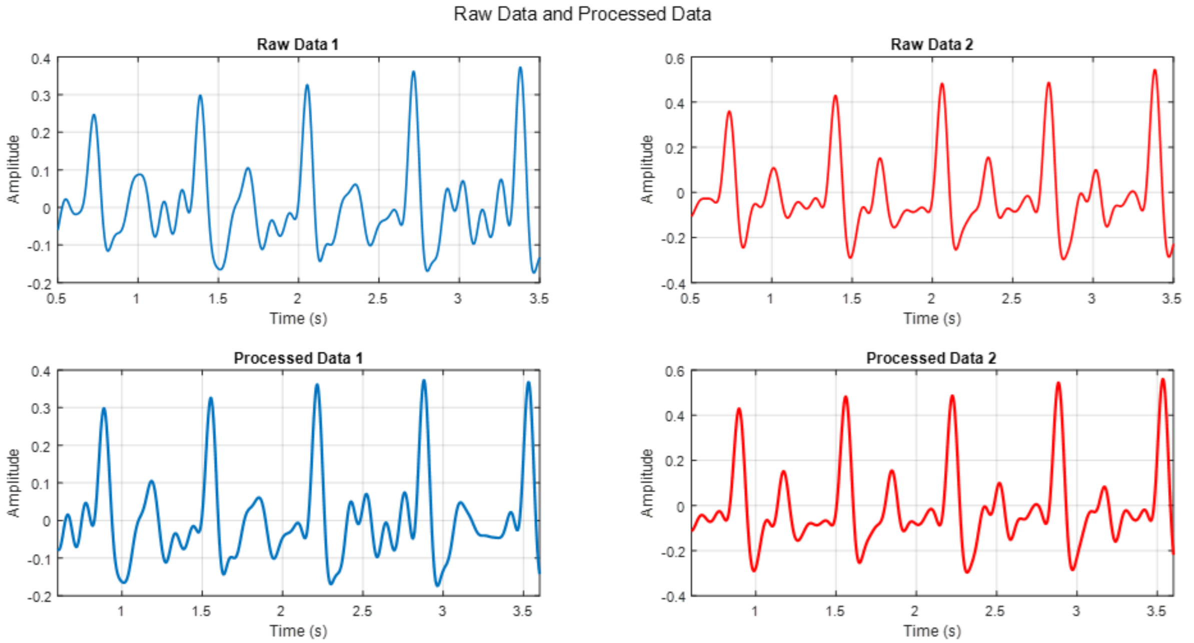

b Reconstructed data from 500 Hz Sampling rate (Time Domain and Frequency Domain)

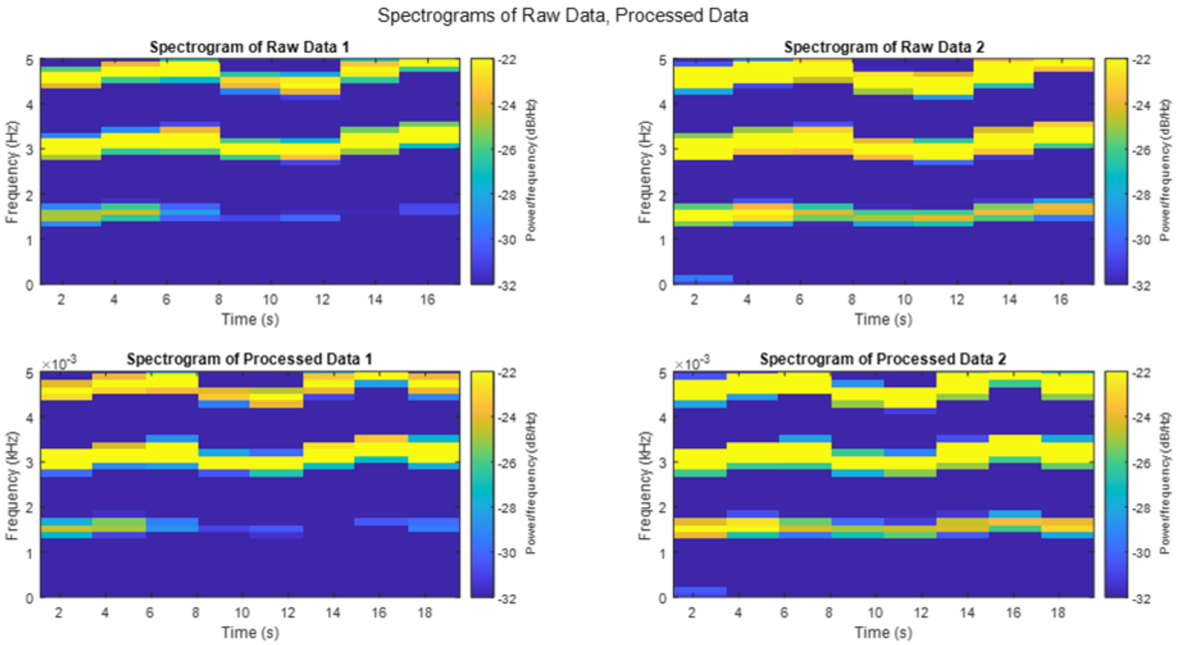

Supplementary Fig.3. Raw signal and reconstructed signal comparison (500 Hz). **a.** Reconstructed data from 500 Hz Sampling rate (Time Domain). **b.** Reconstructed data from 500 Hz Sampling rate (Time Domain and Frequency Domain)

Supplementary Figure 4.

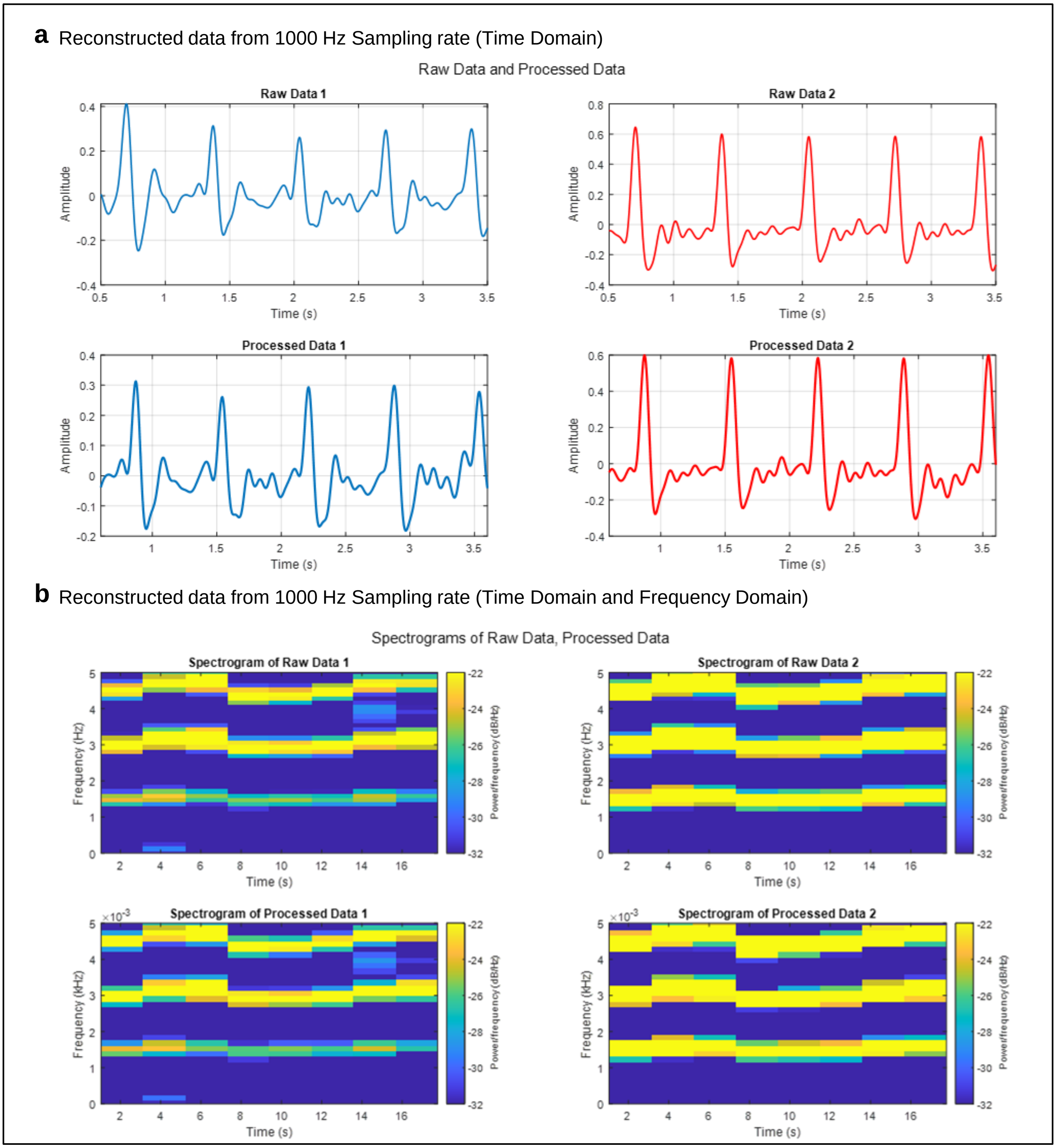

Supplementary Fig.4. Raw signal and reconstructed signal comparison (1000 Hz). **a.** Reconstructed data from 1000 Hz Sampling rate (Time Domain). **b.** Reconstructed data from 1000 Hz Sampling rate (Time Domain and Frequency Domain)

Supplementary Figure 5.

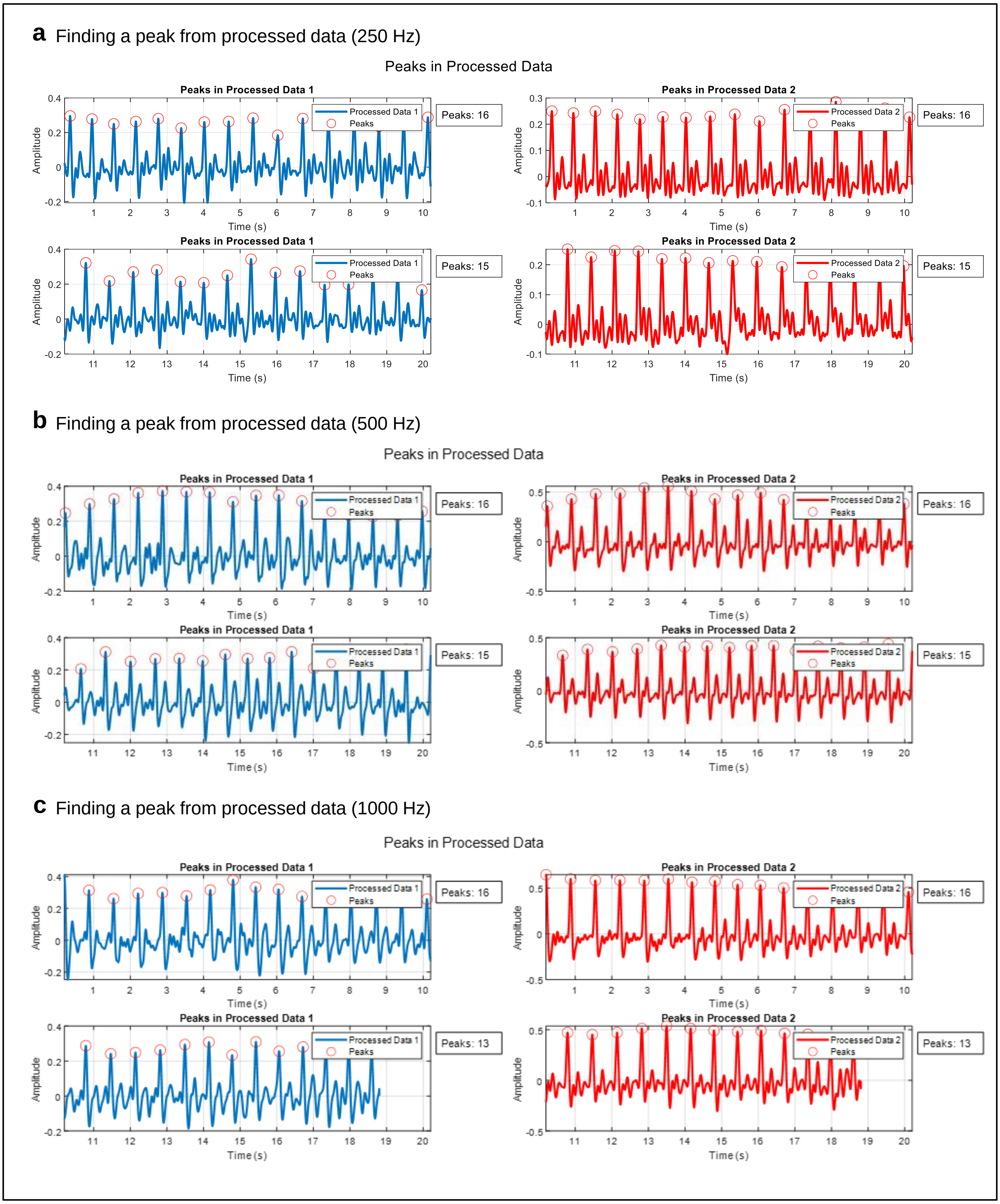

Supplementary Figure 6.

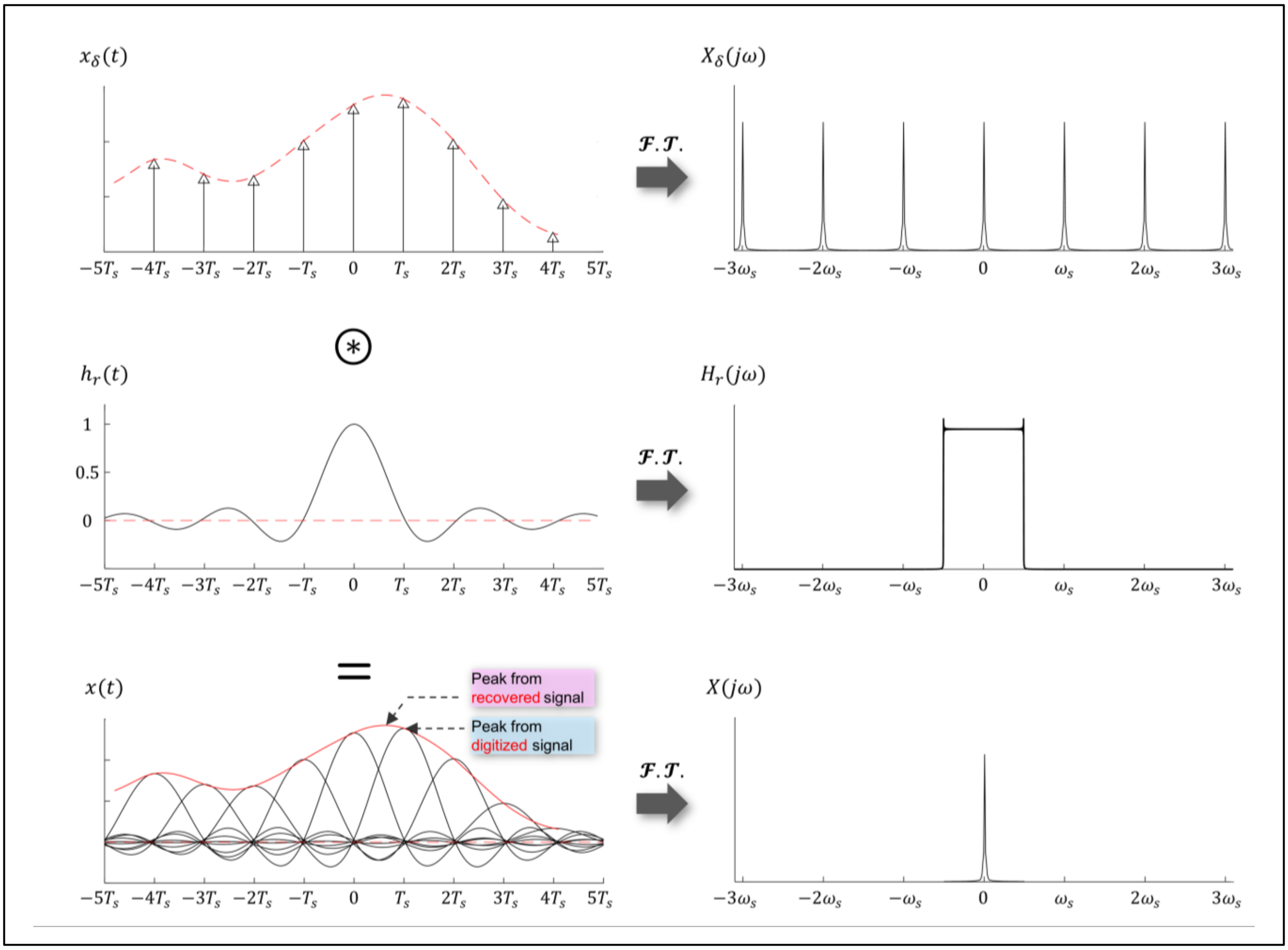

Supplementary Fig.6. Reconstruction of a digitally sampled signal. Analysis in the time domain and frequency domain

Supplementary Figure 7.

a Blood pressure before and after bike

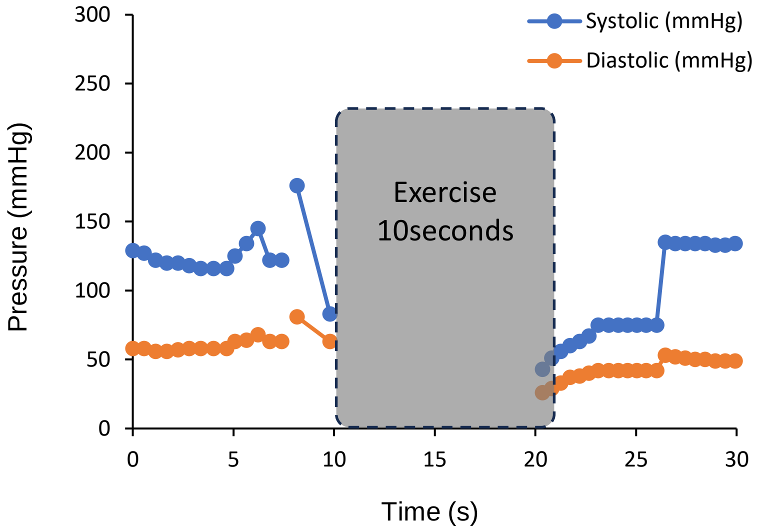

b Heart rate before and after bike

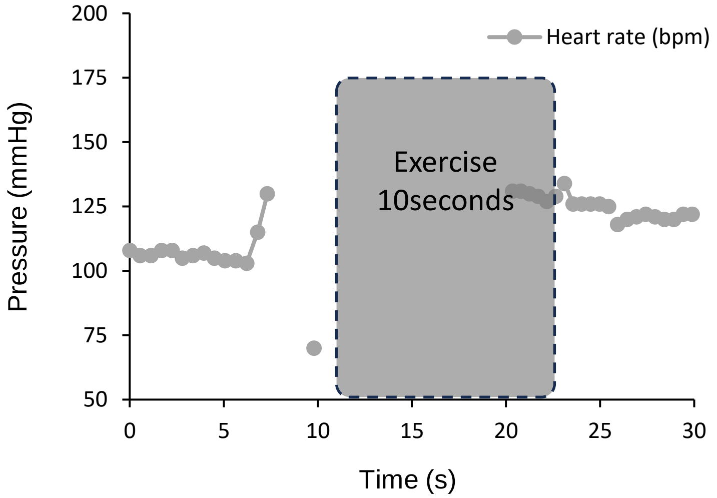

c Summary Table

	Average Systolic pressure (mmHg)	Average Diastolic pressure (mmHg)	Average Heart rate (bpm)
Before bike exercise	124.4375	61.375	100.875
After bike exercise	117.8182	48.091	121.1

Supplementary Information.

To address why the restored and digitized signal have different peaks, we first provide the digitized signal $x[n]$, which is represented as follows.

$$x[n] = x(nT_s)$$

Here, T_s represents the time interval between samples. To represent this discrete-time signal, $x[n]$, as a continuous signal, we can express it as follows by multiplying it with the impulse train.

$$x_\delta(t) = \sum_n x[n] \cdot \delta(t - nT_s)$$

Where, $\delta(t - nT_s)$ is the impulse train with an interval of T_s. The shape of $x_\delta(t)$ in the time domain and frequency domain is shown in the first line of Supplementary Figure 6. From here we can convert it as follows.

$$x_\delta(t) = \sum_n x(nT_s) \cdot \delta(t - nT_s)$$

$$where, \qquad x(nT_s) \cdot \delta(t - nT_s) = x(t) \cdot \delta(t - nT_s)$$

We may write $x_\delta(t)$ as follows.

$$x_\delta(t) = x(t) \cdot p(t)$$

$$Here, \qquad p(t) = \sum_n \delta(t - nT_s)$$

And from here, in order to interpret the signal from the frequency domain, we can use the Fourier Transform of $x_\delta(t)$.

$$X_\delta(j\omega) = \frac{1}{2\pi} X(j\omega) \circledast P(j\omega)$$

$$where, \qquad P(j\omega) = \frac{2\pi}{T_s} \sum_k \delta(\omega - k\omega_s)$$

This can be summarized as follows.

$$X_\delta(j\omega) = \frac{1}{2\pi} X(j\omega) \circledast \frac{2\pi}{T_s} \sum_k \delta(\omega - k\omega_s)$$

$$X_\delta(j\omega) = \frac{1}{T_s} \sum_k X(j(\omega - k\omega_s))$$

A reconstruction filter $H_r(j\omega)$ can be applied here for signal reconstruction. As seen in the frequency domain graph in the second row of Supplementary Figure 6, this function is determined by the sampling rate ($\omega_s = {}^{2\pi}/_{T_s}$). It allows you to reconstruct the desired signal by filtering out the signal from multiple areas in the frequency domain.

$$H_r(j\omega) = \begin{cases} T_s, & |\omega| \le {}^{\omega_s}/_2 \\ 0, & |\omega| > {}^{\omega_s}/_2 \end{cases}$$

To filter out unwanted frequency components, we can multiply $X_\delta(j\omega)$ and the reconstruction function $H_r(j\omega)$ in the frequency domain, which results in convolution in the time domain. This leads to the following equation:

$$X(j\omega) = X_\delta(j\omega) \cdot H_r(j\omega)$$

$$x(t) = x_\delta(t) \circledast h_r(t)$$

$$x(t) = \sum_n x[n] \cdot \delta(t - nT_s) \circledast h_r(t)$$

$$= \sum_n x[n] \cdot h_r(t - nT_s)$$

As seen in Supplementary Figure 6's second row, the $H_r(j\omega)$ appears as a sinc function in the time domain and the equation can be expressed as follows:

$$where, \qquad h_r(t) = \frac{T_s sin(\frac{\omega_s}{2} t)}{\pi t}$$

$$x(t) = \sum_n x[n] \cdot sinc({}^{\omega_s(t - nT_s)}/_{2\pi})$$

The convolution in the time domain between two signals results in the sum of products of sinc functions for each impulse in the sampled signal. This is illustrated in the third row of Supplementary Figure 6. As observed in the frequency domain graph, the desired signal has been reconstructed without any other noise sources. As seen in the figure, it can be observed that the peak points in the digitized signal and the reconstructed signal are different. This occurs when the sum of the sinc functions multiplied at the digitized peak point and the sinc function multiplied at the adjacent point is higher at the midpoint between these two points than the maximum point in the digitized signal. In other words, it's the result of how these sinc functions interact in the convolution process, causing the peak points to differ between the digitized and reconstructed signals. Thus, this increases the accuracy in measuring the peak points of pulses, allowing for a more precise measurement of PWV. It helps obtain a more accurate PWV measurement compared to when the signal hasn't undergone signal processing.
